# Supplementary material for: A Global Screen for Assembly State Changes of the Mitotic Proteome by SEC-SWATH-MS
Source: Cell Syst. 2020 Feb 26;10(2):133–155.e6. doi: 10.1016/j.cels.2020.01.001 (PMC7042714; doi:10.1016/j.cels.2020.01.001)

Q8NF91 | SYNE1_HUMAN | SYNE1 C6orf98 KIAA0796 KIAA1262 KIAA1756 MYNE1
Monomer MW [kDa]: 1011.086 Monomer expected elution fraction: 20

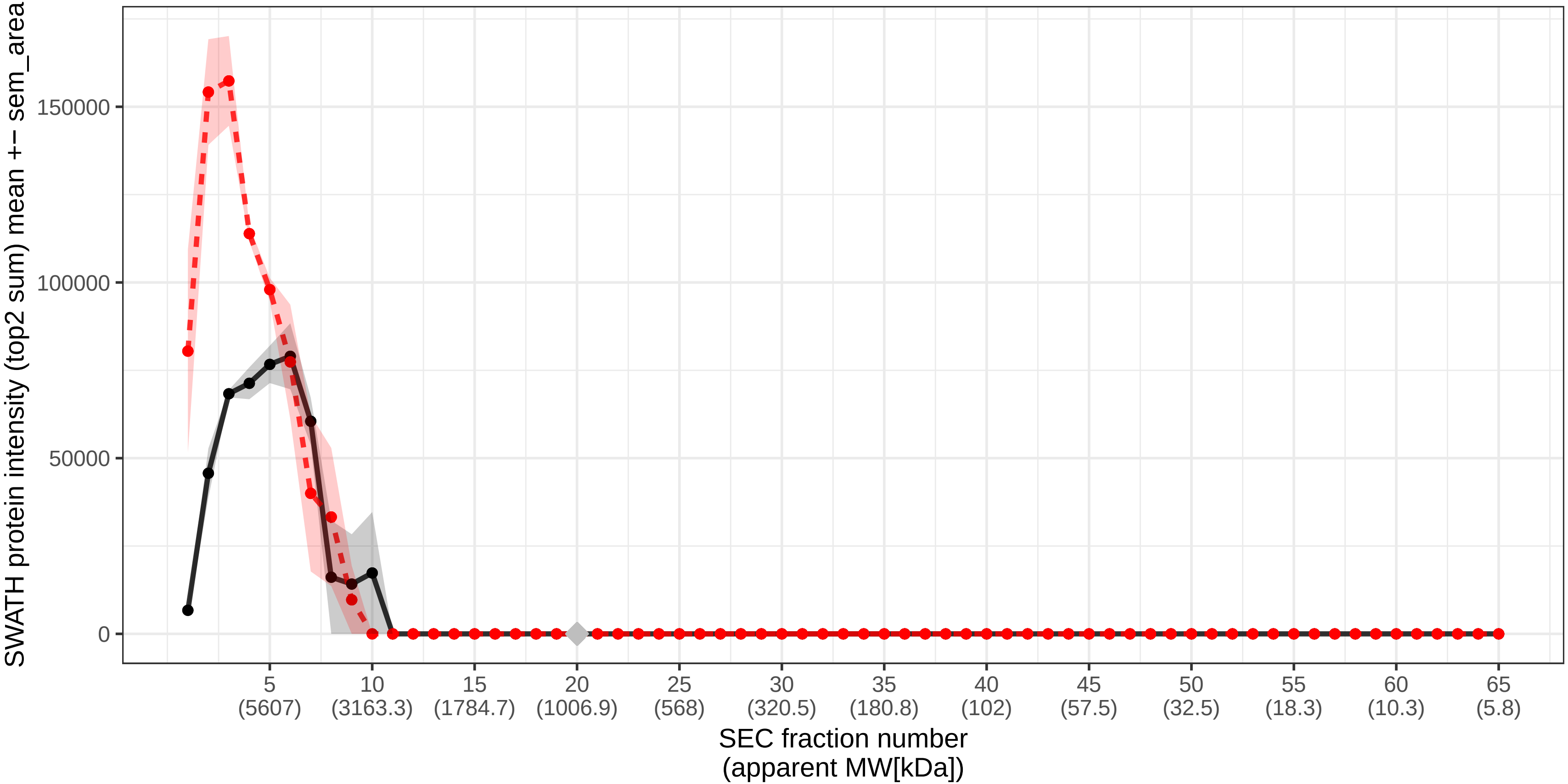

Supplement: Data S1. SEC-SWATH-MS Protein Chromatograms, Related to Figure 1 [file mmc6.zip › SECchrom_Q8NF91_SYNE1_HUMAN_SYNE1_C6orf98_KIAA0796_KIAA1262.pdf]
